# Correction: SARS-CoV-2 and endemic coronaviruses: Comparing symptom presentation and severity of symptomatic illness among Nicaraguan children

**DOI:** 10.1371/journal.pgph.0003480

**Published:** 2024-07-03

**Authors:** Aaron M. Frutos, John Kubale, Guillermina Kuan, Sergio Ojeda, Nivea Vydiswaran, Nery Sanchez, Miguel Plazaola, May Patel, Roger Lopez, Angel Balmaseda, Aubree Gordon

In Table 3, the two risk ratio points estimates are incorrect. The risk ratio for ages 0–4 under ALRI* should be 2.74 (1.04, 7.22) and the risk ratio for ages 10–14 under headache should be 0.72 (0.47, 1.08). Please see the correct Table 3 here.

**Table 3:** Symptom Risk between Endemic HCoV and SARS-CoV-2 by Age Group

**0–4**				
** **	Endemic HCoVs (%)	SARS-CoV-2 (%)	Risk Difference (95% CI)	Risk Ratio (95% CI)
**Measured fever**	218 (50)	44 (75)	-0.24 (-0.36, -0.12)	0.68 (0.57, 0.81)
**Cough**	382 (88)	42 (71)	0.17 (0.05, 0.29)	1.24 (1.05, 1.47)
**Rhinorrhea**	378 (88)	42 (71)	0.16 (0.04, 0.28)	1.23 (1.04, 1.45)
**Congestion**	241 (50)	21 (36)	0.14 (0.09, 0.27)	1.39 (0.97, 1.99)
**Sore throat**	66 (15)	6 (10)	0.05 (-0.03, 0.14)	1.50 (0.68, 3.31)
**Headache**	23 (5)	4 (7)	-0.01 (-0.08, 0.05)	0.79 (0.28, 2.19)
**Loss of appetite**	116 (27)	19 (32)	-0.05 (-0.18, 0.07)	0.83 (0.56, 1.25)
**Diarrhea**	58 (13)	9 (15)	-0.02 (-0.12, 0.08)	0.88 (0.46. 1.68)
**Hospitalized**	17 (4)	4 (7)	-0.03 (-0.10, 0.04)	0.58 (0.20, 1.67)
**Abnormal breathing**	95 (22)	4 (7)	0.15 (0.08, 0.23)	3.24 (1.24, 8.49)
**ALRI**	89 (21)	4 (7)	0.14 (0.6, 0.21)	3.04 (1.16, 7.97)
**ALRI** *	69 (19)	4 (7)	0.12 (0.04,0.19)	2.74 (1.04, 7.22)
**5–9**				
** **	Endemic HCoVs (%)	SARS-CoV-2 (%)	Risk Difference (95% CI)	Risk Ratio (95% CI)
**Measured fever**	39 (35)	12 (41)	-0.06 (-0.26, 0.14)	0.86 (0.52, 1.41)
**Cough**	99 (90)	21 (72)	0.18 (000, 0.35)	1.24 (0.98, 1.57)
**Rhinorrhea**	87 (79)	25 (86)	-0.07 (-0.22, 0.08)	0.92 (0.77, 1.09)
**Congestion**	42 (38)	14 (48)	-0.10 (-0.10, 0.30)	0.79 (0.51, 1.23)
**Sore throat**	52 (47)	12 (41)	0.06 (-0.14, 0.26)	1.14 (0.71, 1.84)
**Headache**	35 (32)	14 (48)	-0.16 (-0.37, 0.04)	0.66 (0.41, 1.04)
**Loss of appetite**	19 (17)	7 (24)	-0.07 (-0.24, 0.10)	0.72 (0.33, 1.54)
**Diarrhea**	4 (4)	3 (10)	-0.07 (-0.18, 0.05)	0.35 (0.08, 1.48)
**Hospitalized**	5 (5)	2 (7)	-0.02 (-0.12, 0.08)	0.66 (0.13, 3.23)
**Abnormal breathing**	10 (9)	2 (7)	0.02 (-0.08, 0.12)	1.32 (0.31, 5.69)
**ALRI**	15 (14)	3 (10)	0.03 (-0.10, 0.16)	1.32 (0.41, 4.25)
**ALRI** *	12 (13)	3 (10)	0.03 (-0.10, 0.16)	1.26 (0.38, 4.16)
**10–14**				
** **	Endemic HCoVs (%)	SARS-CoV-2 (%)	Risk Difference (95% CI)	Risk Ratio (95% CI)
**Measured fever**	15 (28)	12 (36)	-0.08 (-0.28, 0.12)	0.78 (0.42, 1.45)
**Cough**	43 (81)	22 (67)	0.14 (-0.05, 0.34)	1.22 (0.93, 1.60)
**Rhinorrhea**	40 (75)	26 (79)	-0.03 (-0.21, 0.15)	0.96 (0.76, 1.21)
**Congestion**	20 (38)	10 (30)	0.07 (-0.13, 0.28)	1.25 (0.67, 2.32)
**Sore throat**	27 (51)	18 (55)	-0.04 (-0.25, 0.18)	0.93 (0.62, 1.41)
**Headache**	23 (43)	20 (61)	-0.17 (-0.39, 0.04)	0.72 (0.47, 1.08)
**Loss of appetite**	7 (13)	5 (15)	-0.02 (-0.17, 0.13)	0.87 (0.30, 2.52)
**Diarrhea**	2 (4)	2 (6)	-0.02 (-0.12, 0.07)	0.62 (0.09, 4.21)
**Hospitalized**	1 (2)	0	0.02 (-0.02, 0.06)	-
**Abnormal breathing**	3 (6)	3 (9)	-0.03 (-0.15, 0.08)	0.62 (0.13, 2.90)
**ALRI**	3 (4)	0	0.06 (-0.01, 0.12)	-
**ALRI** *	3 (6)	0	0.06 (-0.01, 0.13)	-

*Excluding influenza A, influenza B, RSV, and HMpV coinfections
