# Supplementary figures and images for: SARS-CoV-2 and endemic coronaviruses: Comparing symptom presentation and severity of symptomatic illness among Nicaraguan children
Source: PLOS Glob Public Health. 2022 May 25;2(5):e0000414. doi: 10.1371/journal.pgph.0000414 (PMC9245908; doi:10.1371/journal.pgph.0000414)

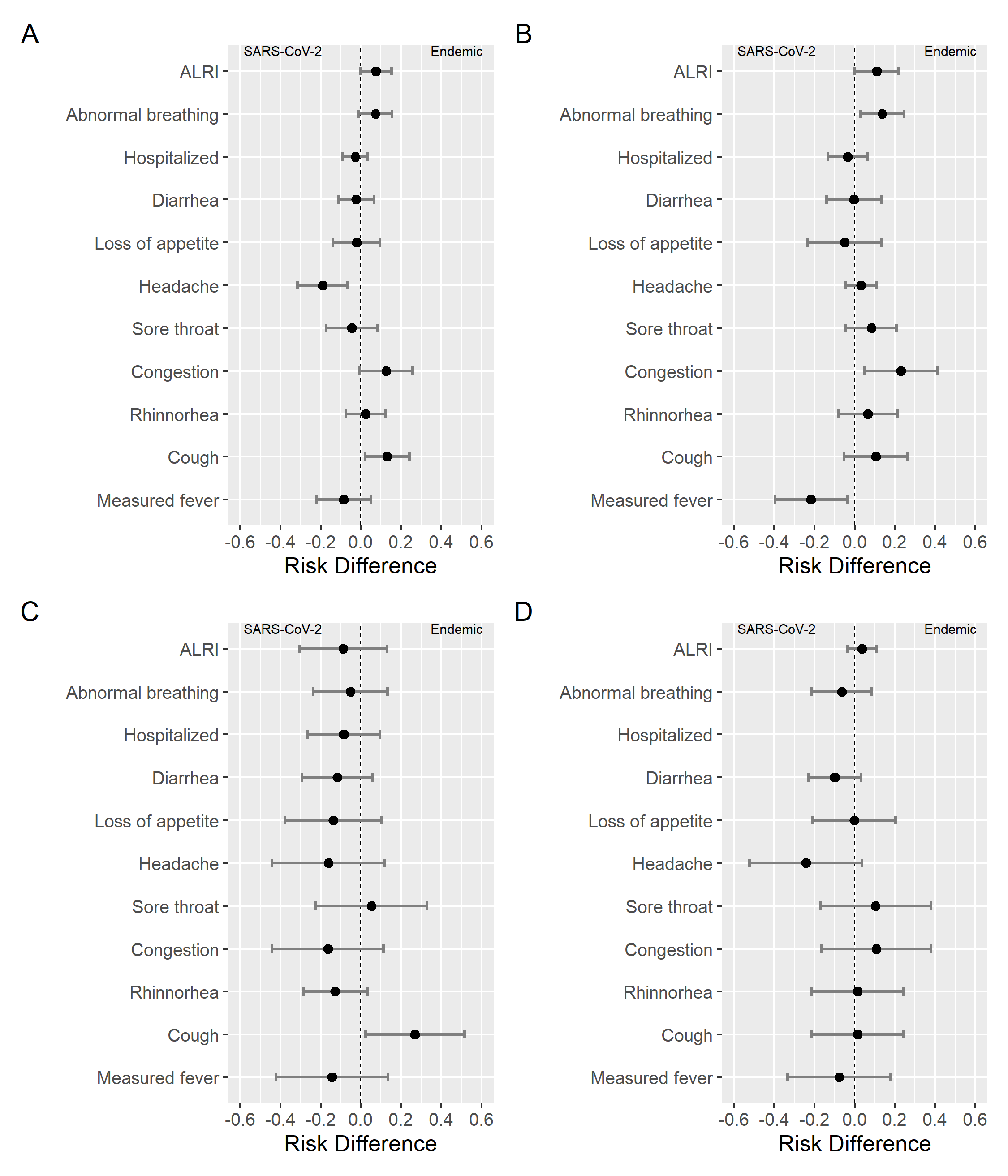

Supplement: S1 Fig — A: All participants. B: Ages 0–4. C: Ages 5–9. D Ages: 10–14. (TIFF) [file pgph.0000414.s001.tiff]

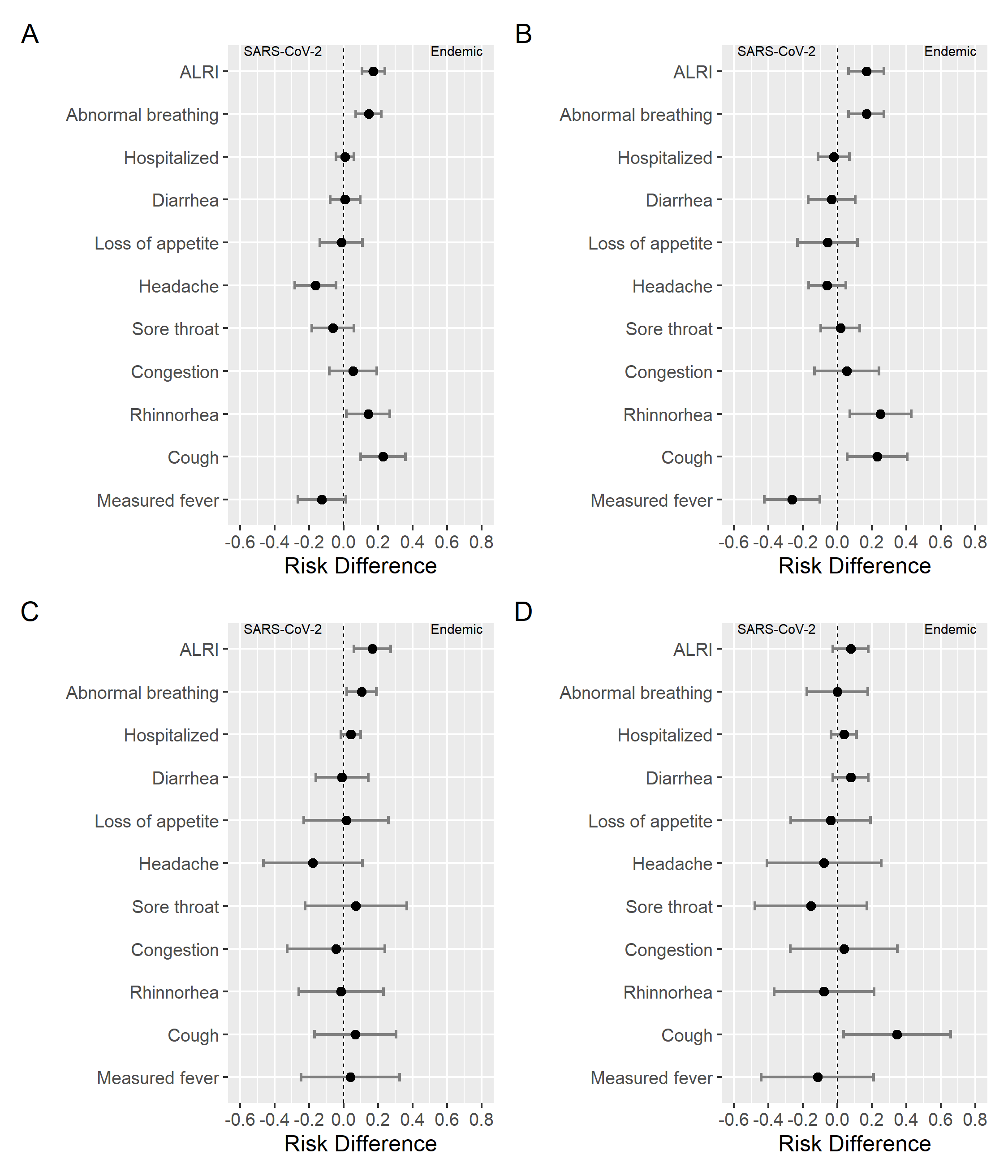

Supplement: S2 Fig — A: All participants. B: Ages 0–4. C: Ages 5–9. D Ages: 10–14. (TIFF) [file pgph.0000414.s002.tiff]

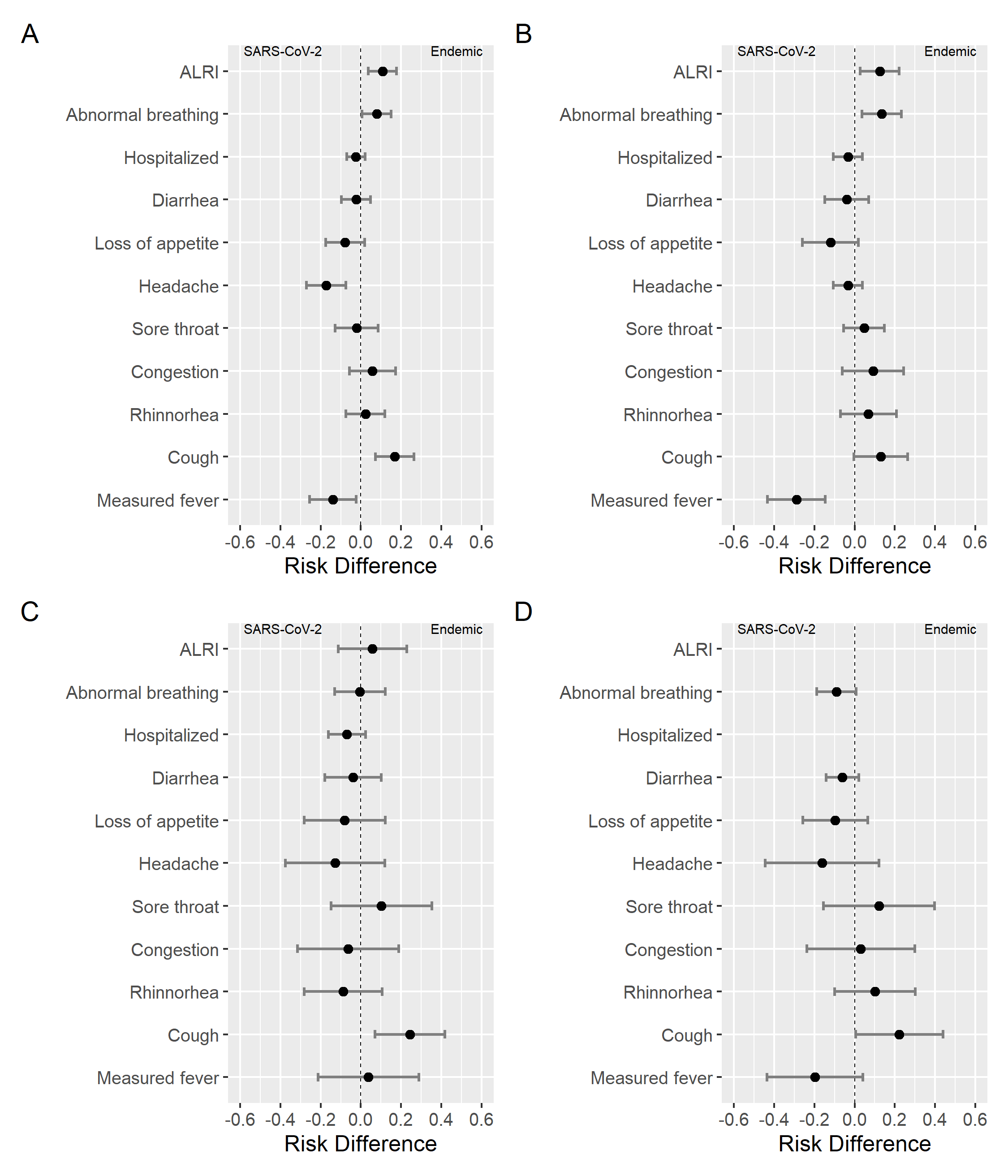

Supplement: S3 Fig — A: All participants. B: Ages 0–4. C: Ages 5–9. D Ages: 10–14. (TIFF) [file pgph.0000414.s003.tiff]

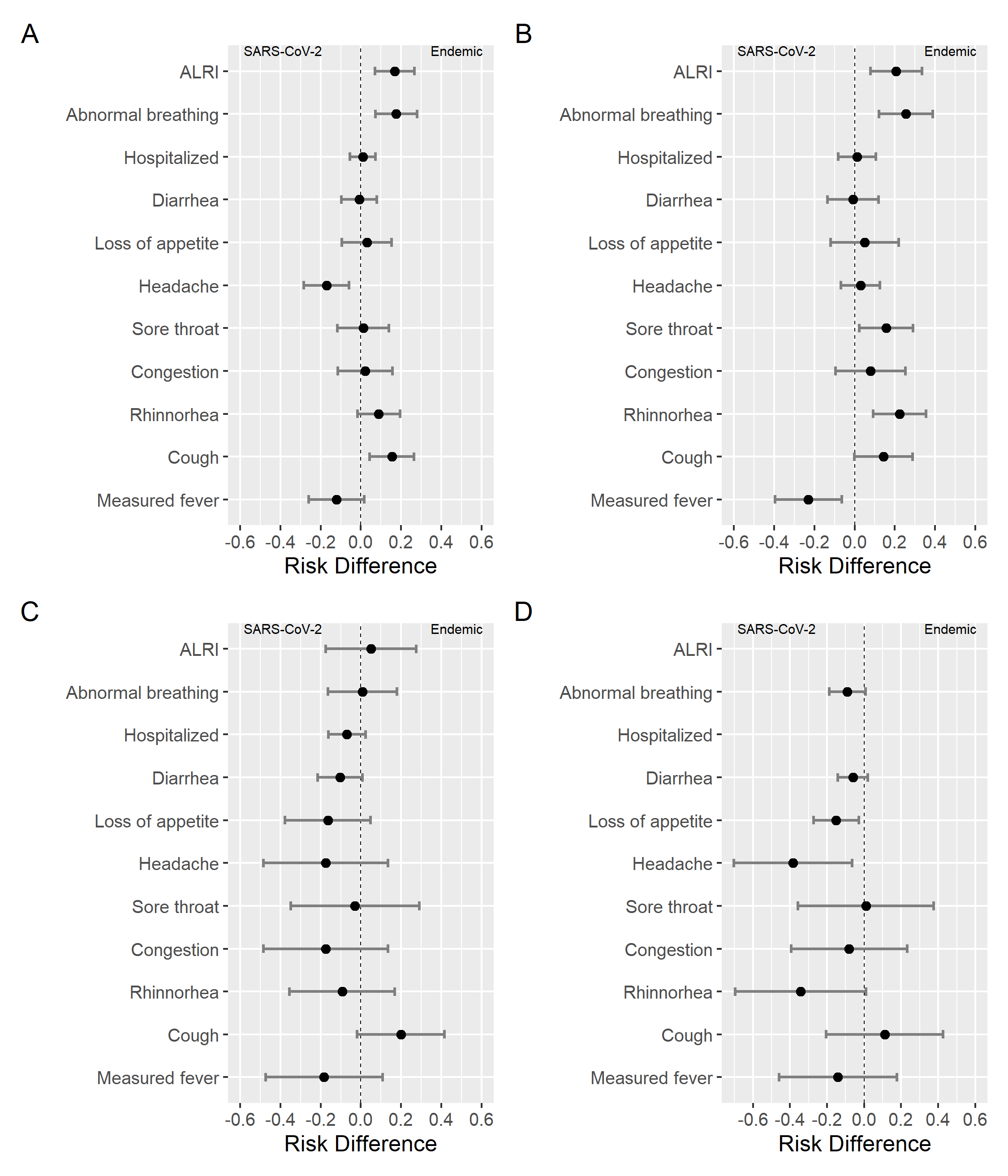

Supplement: S4 Fig — A: All participants. B: Ages 0–4. C: Ages 5–9. D Ages: 10–14. (TIFF) [file pgph.0000414.s004.tiff]

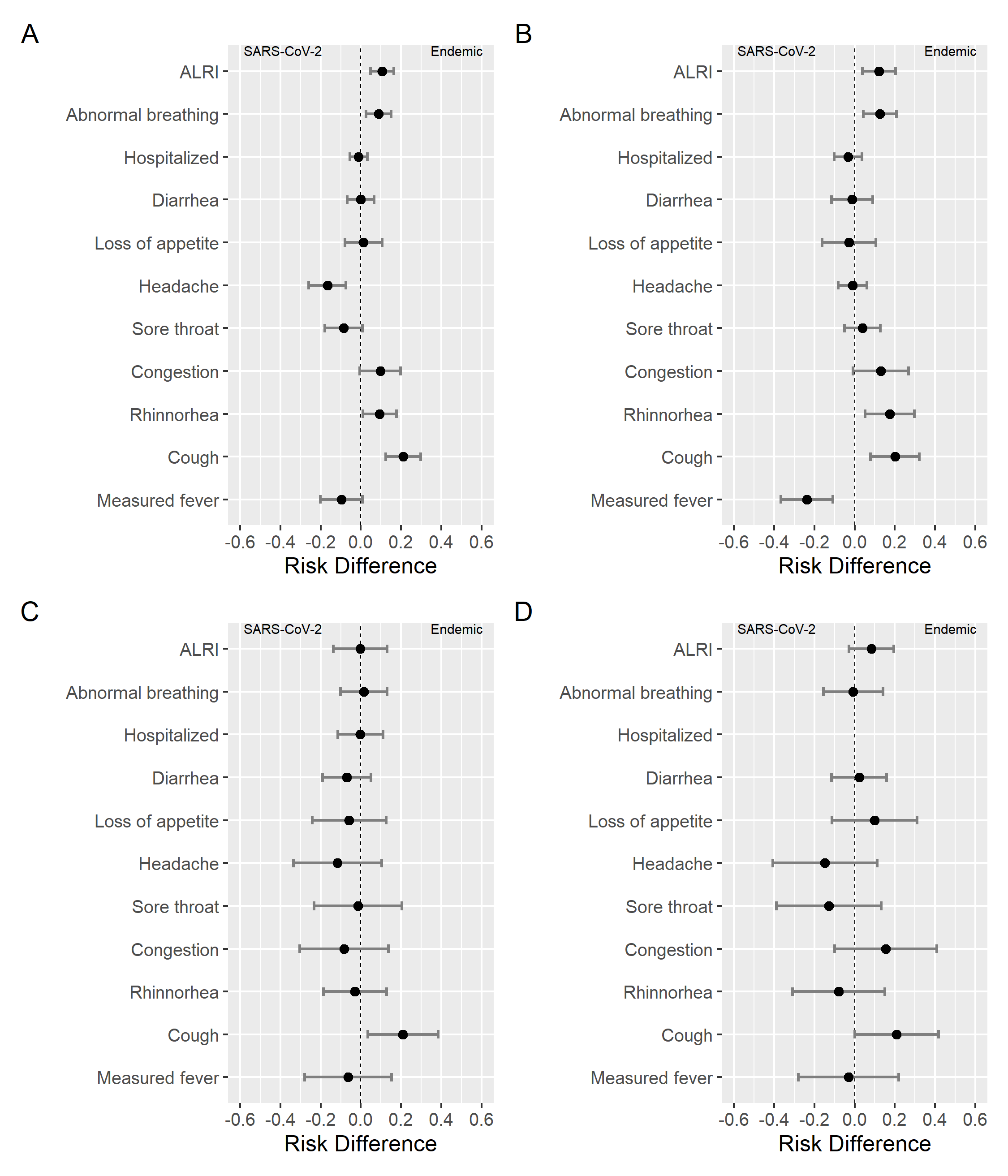

Supplement: S5 Fig — A: All participants. B: Ages 0–4. C: Ages 5–9. D Ages: 10–14. (TIFF) [file pgph.0000414.s005.tiff]

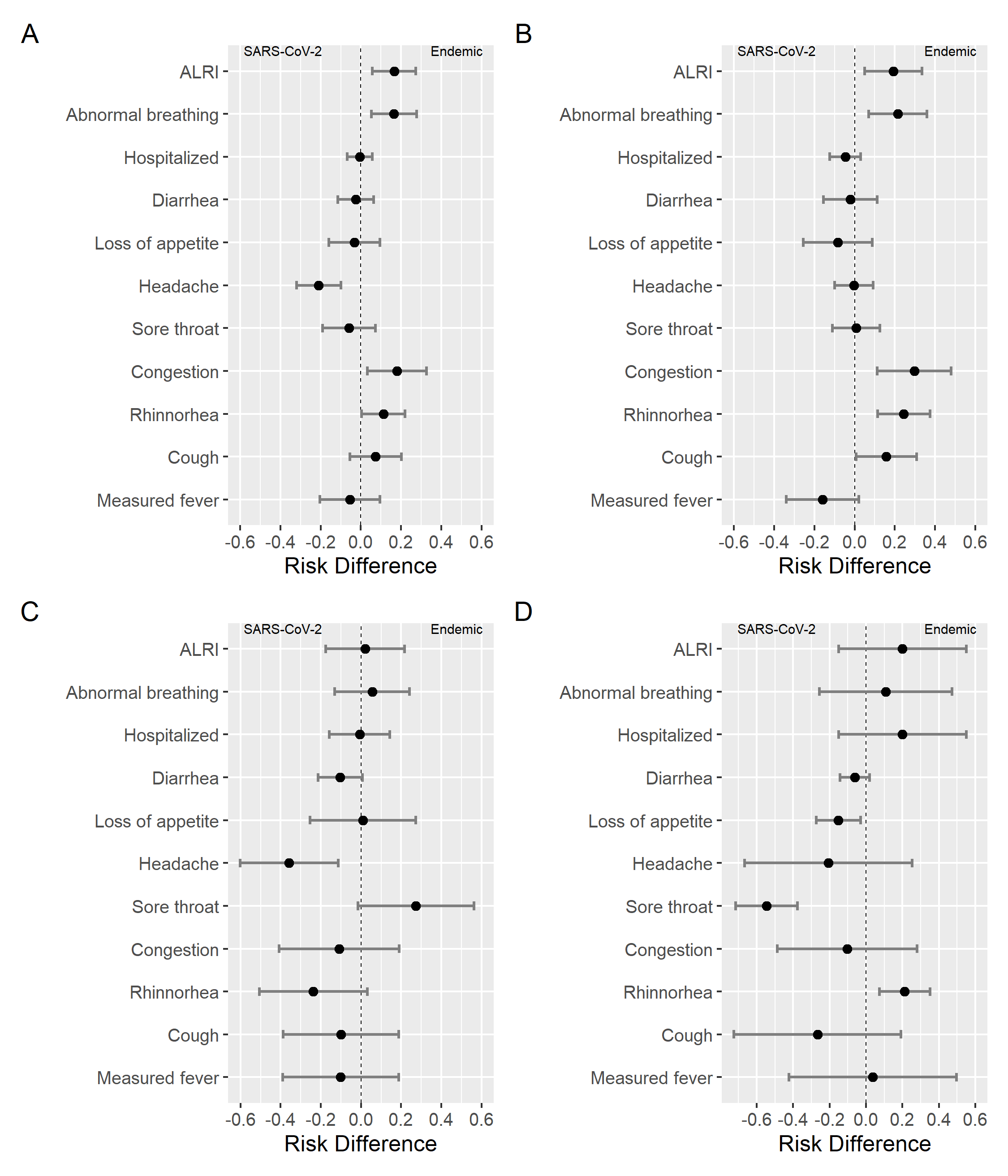

Supplement: S6 Fig — A: All participants. B: Ages 0–4. C: Ages 5–9. D Ages: 10–14. (TIFF) [file pgph.0000414.s006.tiff]
