# Supplementary material for: SARS-CoV-2 and endemic coronaviruses: Comparing symptom presentation and severity of symptomatic illness among Nicaraguan children
Source: PLOS Glob Public Health. Author manuscript; Available in PMC 2022 Jun 30. (PMC9245908; doi:10.1371/journal.pgph.0000414)
Supplement: Supplementary file 1 [file NIHMS1813892-supplement-Supplementary_Figures.pdf]

Supporting information

S1 Fig. Symptom Risk Difference between Endemic HCoVs and SARS-CoV-2 for Females.

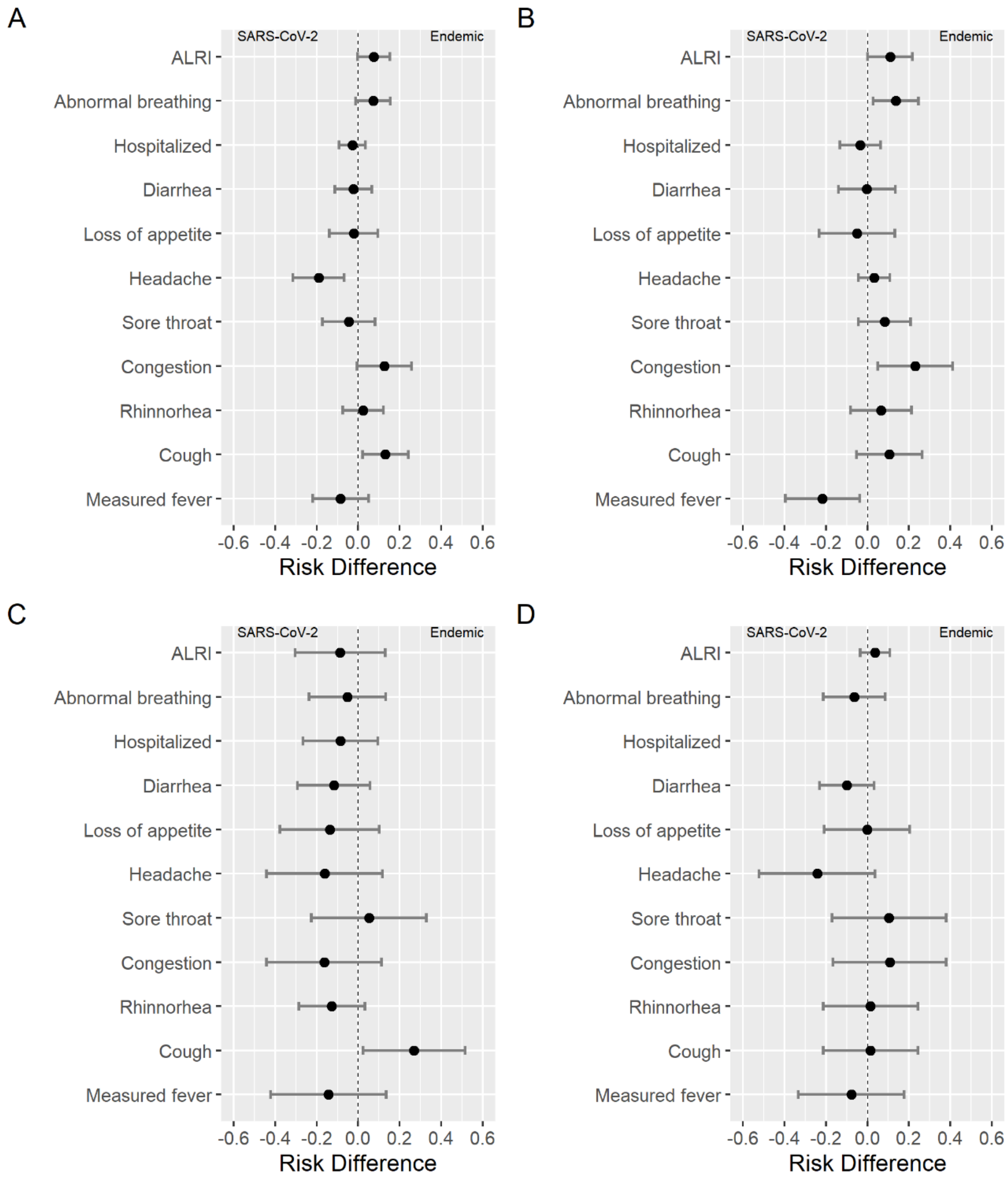

A: All participants. B: Ages 0-4. C: Ages 5-9. D Ages: 10-14.

S2 Fig. Symptom Risk Difference between Endemic HCoVs and SARS-CoV-2 for Males.

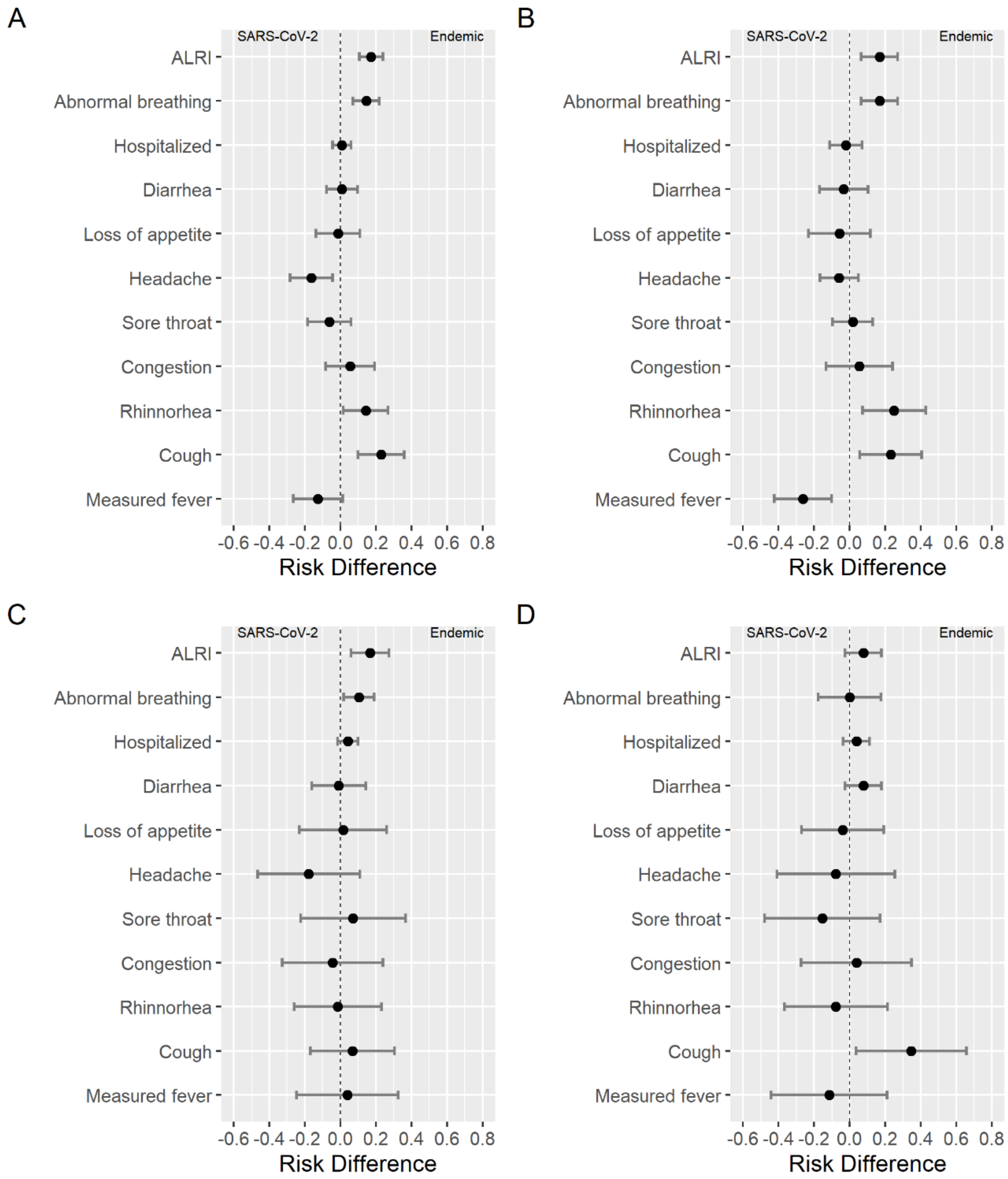

A: All participants. B: Ages 0-4. C: Ages 5-9. D Ages: 10-14.

S3 Fig. Symptom Risk Difference NL63 and SARS-CoV-2.

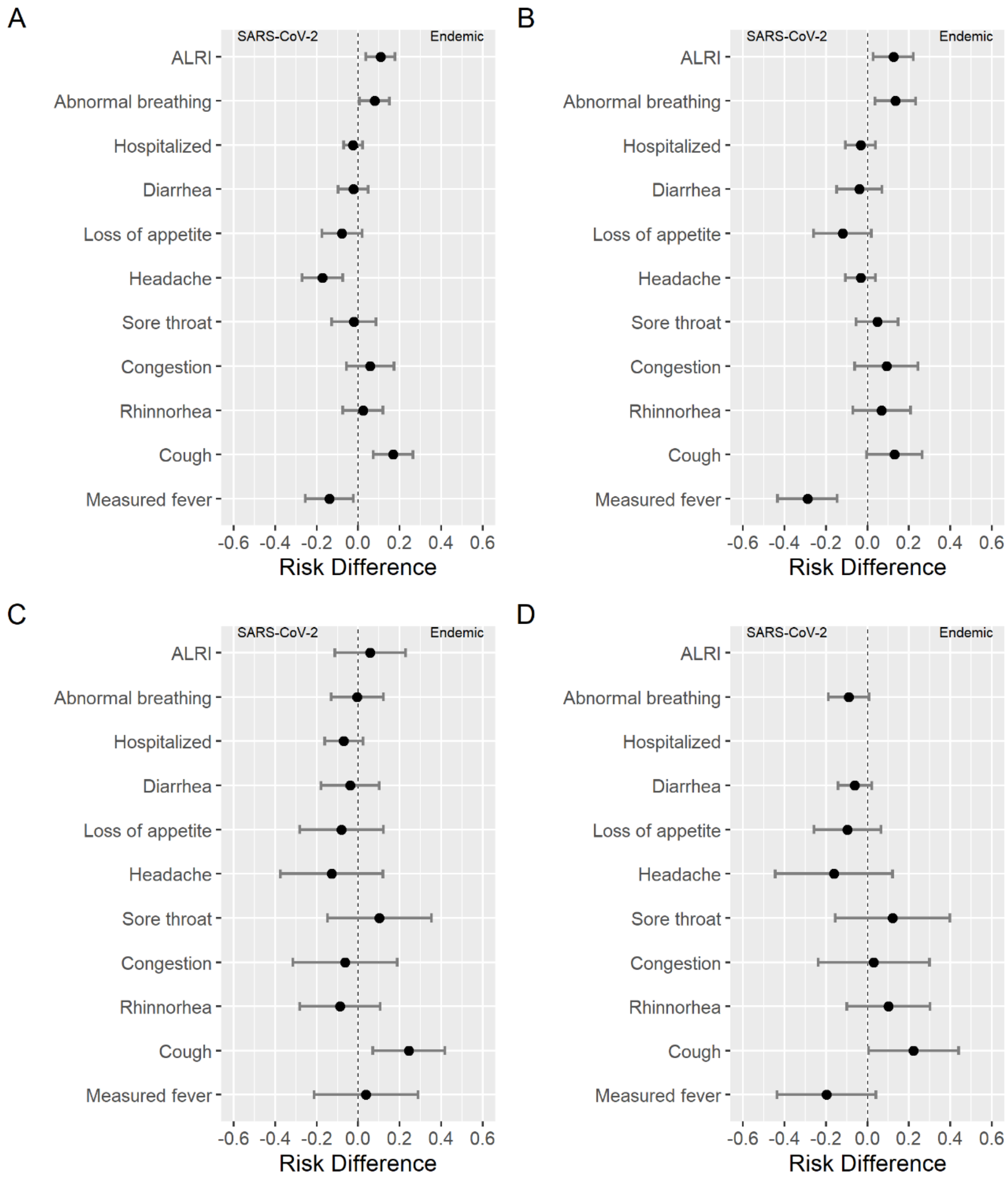

A: All participants. B: Ages 0-4. C: Ages 5-9. D Ages: 10-14.

S4 Fig. Symptom Risk Difference between 229E and SARS-CoV-2.

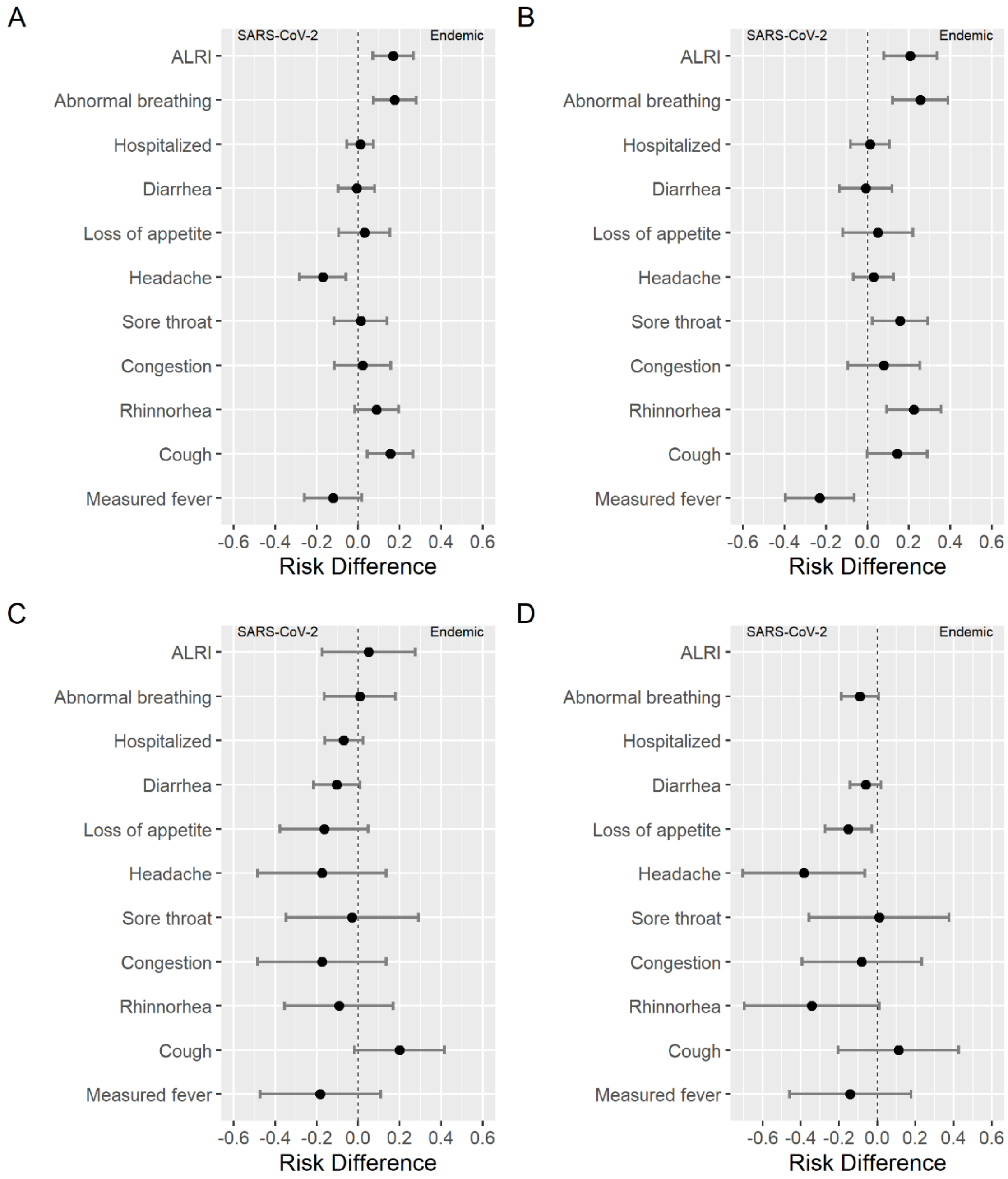

A: All participants. B: Ages 0-4. C: Ages 5-9. D Ages: 10-14.

S5 Fig. Symptom Risk Difference between OC43 and SARS-CoV-2.

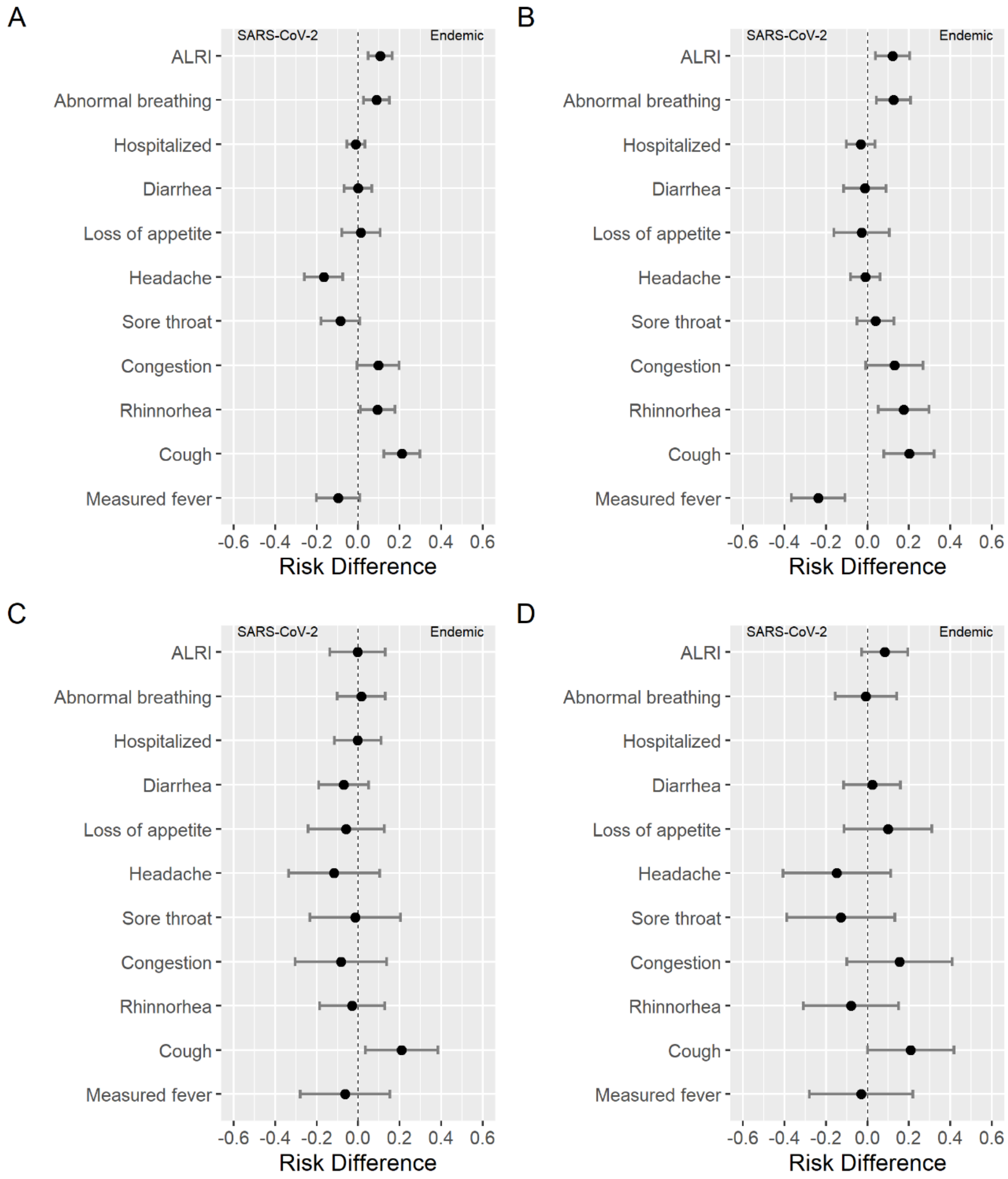

A: All participants. B: Ages 0-4. C: Ages 5-9. D Ages: 10-14.

S6 Fig. Symptom Risk Difference between HKU1 and SARS-CoV-2.

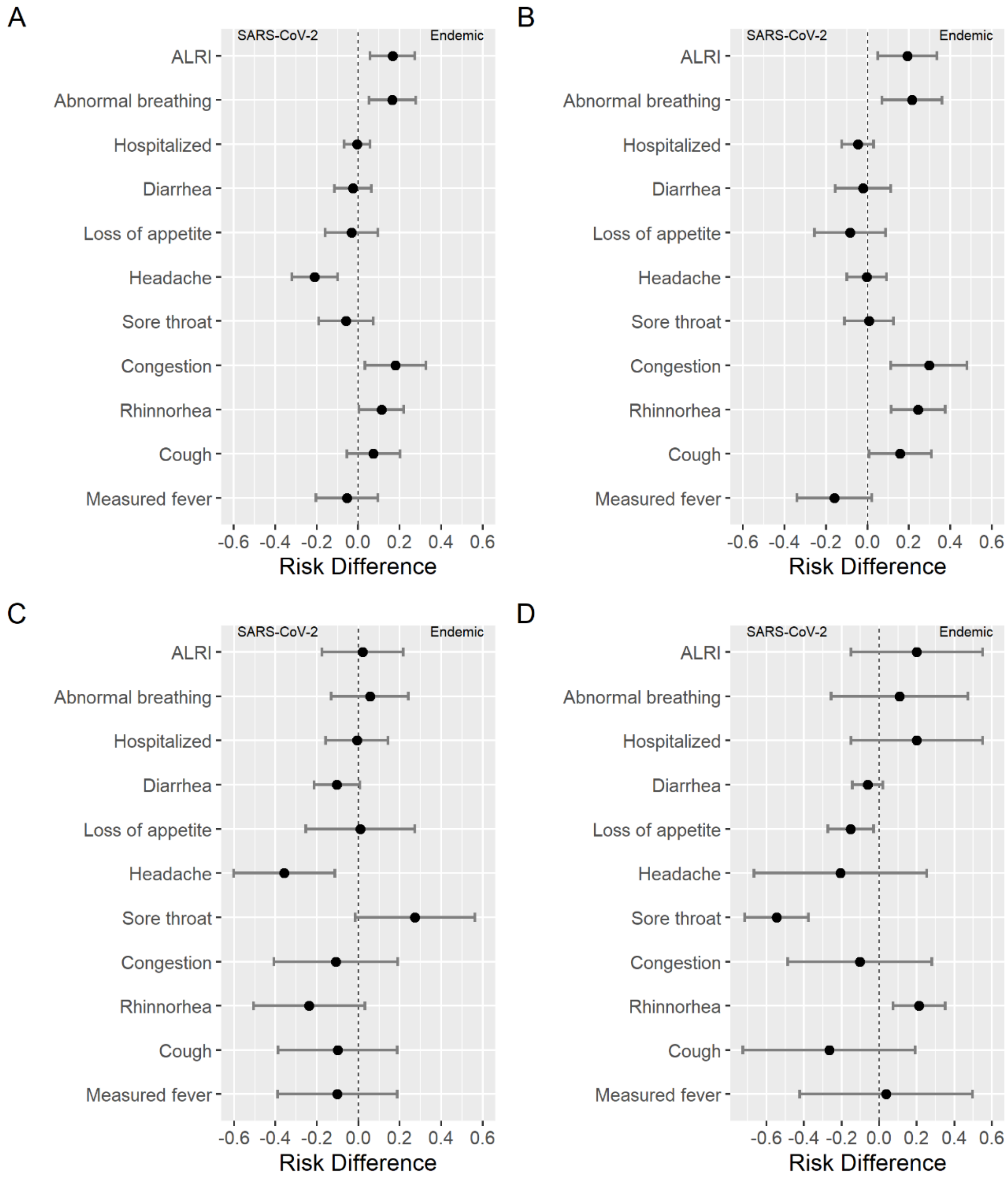

A: All participants. B: Ages 0-4. C: Ages 5-9. D Ages: 10-14.
